# Evaluating the potential impact of lifestyle-based behavior change interventions delivered at the time of colorectal cancer screening

**DOI:** 10.1007/s10552-023-01773-0

**Published:** 2023-11-05

**Authors:** Veeraj Shah, Greta Geller, Diane Xu, Lily Taylor, Simon Griffin, Juliet A. Usher-Smith

**Affiliations:** 1 Primary Care Unit, Department of Public Health and Primary Care, University of Cambridge, Forvie Site, Robinson Way, Cambridge, CB2 0SR UK; 2 School of Clinical Medicine, University of Cambridge, Addenbrooke’s Hospital, Hills Rd, Cambridge, CB2 0SP UK

**Keywords:** Colorectal cancer, Screening, Lifestyle behaviors, Behavior change, Behavior change techniques

## Abstract

**Purpose:**

To analyze interventions implemented at the time of colorectal cancer (CRC) screening, or among individuals who have previously undergone investigation for CRC, focused on reducing CRC risk through promotion of lifestyle behavior change. Additionally, this review evaluated to what extent such interventions apply behavior change techniques (BCTs) to achieve their objectives.

**Methods:**

Five databases were systematically searched to identify randomized control trials seeking to reduce CRC risk through behavior change. Outcomes were changes in health-related lifestyle behaviors associated with CRC risk, including changes in dietary habits, body mass index, smoking behaviors, alcohol consumption, and physical activity. Standardized mean differences (SMDs) with 95% confidence intervals (CIs) were pooled using random effects models. BCT’s were coded from a published taxonomy of 93 techniques.

**Results:**

Ten RCT’s met the inclusion criteria. Greater increase in fruit/vegetable consumption in the intervention group were observed with respect to the control (SMD 0.13, 95% CI 0.08 to 0.18; *p* < 0.001). Across fiber, alcohol, fat, red meat, and multivitamin consumption, and smoking behaviors, similar positive outcomes were observed (SMD 0.09–0.57 for all, *p* < 0.01). However, among physical activity and body mass index, no difference between the intervention groups compared with controls were observed. A median of 7.5 BCTs were applied across included interventions.

**Conclusion:**

While magnitude of the observed effect sizes varied, they correspond to potentially important changes in lifestyle behaviors when considered on a population scale. Future interventions should identify avenues to maximize long-term engagement to promote sustained lifestyle behavior change.

## Introduction

Colorectal cancer (CRC) is the 3rd most common cancer globally and the 4th most common cancer in the United Kingdom, with approximately 100 newly diagnosed cases each day in the UK [1]. Current research attributes approximately 40% of CRC risk to body mass index and lifestyle factors such as physical activity, diet, smoking, and alcohol consumption [1]. As such, the risk of colorectal cancer can be reduced by modifying exposure to such lifestyle risk factors through reducing physical inactivity, being overweight or obese, and consumption of an unhealthy diet [2–4].

To reduce CRC incidence and mortality, many countries have established national colorectal cancer screening programs [5]. The English colorectal cancer screening program, started in 2006, has been associated with a 15% reduction in colorectal cancer mortality [6]. A growing body of literature describes colorectal cancer screening as a “teachable moment” to facilitate lifestyle-related behavior change that may reduce future CRC risk [7]. Managing these risk factors has also been shown to reduce the risk of developing other chronic conditions, including cardiovascular disease and type II diabetes mellitus.

A number of studies have delivered individual-level interventions at the time of colorectal cancer screening focused on reducing CRC risk via improving adherence to several lifestyle behaviors, including physical activity, diet, smoking, and alcohol consumption. These studies have shown different degrees of success in changing behavior [8–10]. A recent systematic review by *Orange et al.* synthesized the evidence on physical activity and dietary interventions among individuals attending colorectal and breast cancer screening [11]. However, that review does not address several other lifestyle behaviors associated with CRC risk, and excludes interventions delivered outside of the context of CRC screening that could feasibly be delivered within. Additionally, that review does not assess the application of behavior change techniques (BCTs) within existing interventions aimed at promoting positive changes of these specific cancer-preventive lifestyle behaviors. This is especially important given the utility of BCTs in identifying specific components of interventions that may be associated with behavior change, and to characterize behavior change focused interventions in general.

The goal of this review is to systematically analyze interventions implemented at the time of CRC screening, or among individuals who have previously undergone investigation for CRC, that are focused on reducing CRC risk through promotion of lifestyle behavior change. An additional goal of this review is to evaluate to what extent such interventions apply BCTs to achieve their objectives.

## Methods

**Fig. 1:**
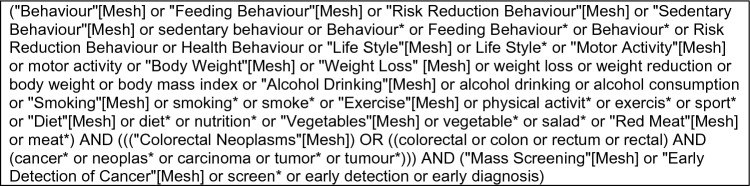
Summary of the search terms used in our database search for studies

This review was prospectively registered on the PROSPERO register of systematic reviews (CRD42021287850) and followed the Preferred Reporting Items for Systematic Reviews and Meta-Analyses (PRISMA) guidelines. An electronic search of CENTRAL, Embase, Medline, PyscInfo, and Web of Science was performed in November 2021 using the search strategy shown in Fig. 1. Our search strategy utilized Boolean operators of AND/OR to concatenate search terms. Search string syntax was adapted to different databases as necessary, and dates were not restricted. We also manually searched reference lists of included publications to identify potentially eligible studies.

### Inclusion/exclusion criteria

Original research articles were included if: (1) the article was available in English; (2) the study was a randomized controlled trial; (3) participants were ≥ 18 years attending a population-based cancer screening program for colorectal cancer OR have previously undergone investigations for colorectal cancer; (4) an individual-level intervention was delivered that addressed one or more of the following colorectal-cancer preventive lifestyle behaviors: physical activity, diet (e.g., red meat or fruit/vegetable consumption, fat consumption), smoking, and alcohol consumption; (5) the intervention was implemented at the time of colorectal cancer screening, or explicitly focused on the reduction of colorectal cancer risk through behavior change; and (6) the study has a control group that either does not receive an intervention or receives treatment distinctly different from the intervention group.

Articles were excluded if they were: (1) not available in English; (2) qualitative studies, literature reviews, or protocol papers; (3) interventions that sought to affect either attendance or participation in colorectal cancer screening programs; (4) interventions that did not seek to change lifestyle-related behaviors such as physical activity, diet, smoking, and alcohol consumption; (5) interventions measuring changes in lifestyle behaviors through qualitative analysis; and (6) interventions that did not have a control group.

### Outcomes

In this review, outcomes of interest are changes in health-related lifestyle behaviors associated with risk of colorectal cancer incidence between baseline and last available follow-up among eligible intervention participants. This includes changes in dietary habits, body mass index (BMI), smoking behaviors, alcohol consumption, and physical activity from intervention baseline to the last available follow-up. Each of these outcomes are risk factors associated with the prevention of CRC [12, 13]. We excluded outcomes focused on screening participation, adherence, attendance, engagement, or screening-related behavioral interventions with no concrete behavioral change action.

### Study selection

Identified studies from the search databases were downloaded into Endnote and exported to Rayyan. The titles and abstracts of a random selection of 10% of the papers were independently assessed by two or more members of the review team against the listed inclusion and exclusion criteria (VS, GG, DX). After review and discussion, the remaining 90% of titles and abstracts were assessed by two members of the review team, with disagreements being resolved in the presence of a third reviewer (VS, GG, DX). Studies whose title and abstract were not relevant were rejected, and those that cannot be categorized definitively underwent a full text review.

Next, three reviewers (VS, GG, DX) assessed full-text articles against inclusion and exclusion criteria and met regularly to discuss results. Papers that did not meet criteria at this stage will were also excluded. Throughout the selection process, reviewers met regularly to discuss findings and any irregularities observed. Any disagreements during full-text review and data extraction were resolved in the presence of a third reviewer (SG, JUS).

### Data extraction

The following data fields were extracted from all studies meeting inclusion criteria: study design, information on participants, information on the intervention (including the components within the TIDieR checklist), behavior change techniques applied during intervention, intervention outcomes, and results [14]. Where studies followed up participants at multiple time points, we extracted data from the longest available follow-up. A data extraction template was built using Microsoft Excel and was piloted by 3 researchers with a small set of full-text articles to resolve any conflicts. Data extraction was completed by a primary reviewer and checked by a secondary reviewer. As such, data extraction for each paper was reviewed by a minimum of two authors.

Behavior change techniques were coded using the *Michie *et al*.* taxonomy of 93 BCTs [15]. Two individual pilots for BCT coding were done, where in each pilot one publication was coded by three authors each (VS, GG, DX). This was completed to establish consensus in coding procedures and familiarity with the *Michie *et al*.* taxonomy. The remaining publications were coded independently by two authors, with a third author resolving any disagreements (VS, GG, DX).

### Risk of bias

Risk of bias was examined using the Cochrane Risk of Bias tool (ROB2), and all studies were examined by two independent reviewers as per Cochrane recommendations [16]. We focused on the following domains pertaining to bias: bias arising from the randomization process; bias due to deviations from intended interventions; bias due to missing outcome data; bias in measurement of the outcome; bias in selection of the reported result.

For each study, the ROB2 tool was used to assess risk of bias using the study’s primary outcome. For each domain, a series of signaling questions (Yes; Probably yes; Probably no; No; No information) were applied to conclude judgements for each domain. The overall risk of bias was expressed as ‘low,’ ‘high,’ or ‘some concerns.’

### Data synthesis

Data from the selected interventions were characterized using the TIDieR checklist and coded using the specific BCTs they apply [14]. Regarding measures of treatment effect, measures of primary outcomes (physical activity, diet, alcohol consumption, smoking) were analyzed separately. For interventions with outcome data presented after the intervention concludes, outcomes were reported for the longest follow-up period.

Effect sizes were summarized using forest plots. Effect sizes for dichotomous data were expressed as odds ratios and for continuous outcomes were converted to standardized mean differences (SMD) to account for differences in measurement strategies between interventions. When data for the same outcome were provided as dichotomous data by some studies and as continuous data by others, log odds ratios were converted to SMDs using the methods outlined by *Chinn* [17].

We combined SMDs across studies for each specific outcome using inverse variance-weighted meta-analysis with each study providing a single effect estimate. Effect sizes were pooled with 95% confidence intervals using a random effects model, and heterogeneity was assessed via a chi-squared test and the I^2^ statistic. For the I^2^ statistic, we considered a value of 50% or greater to represent substantial heterogeneity. No meta-regressions were performed due to a low number of available studies for each outcome represented, as per the Cochrane Handbook’s recommendations [18, 19].

## Results

**Fig. 2:**
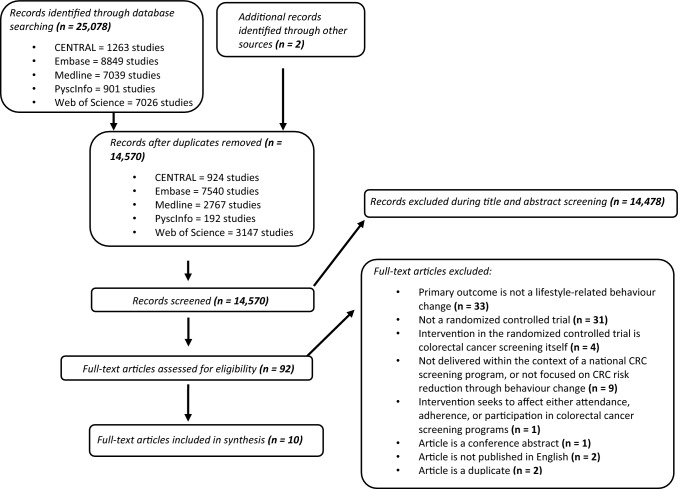
PRISMA diagram describing the number of studies included in each stage of the screening process

We screened 25,087 titles/abstracts for initial eligibility, of which 92 full-text articles met the criteria for additional screening. From this, a total of 10 articles met all inclusion criteria and are included in this review (Fig. 2).

Table 1 provides the details of the included studies, with a focus on the interventions delivered. Of the included studies, six took place in the context of a national CRC screening program [8–10, 20–22], two took place in the context of screening programs at private hospitals/clinics [23, 24], and two occurred through the context of workplaces [25, 26]. Three of the ten studies included populations that had received/undergone previous investigations for CRC, primarily removal of bowel polyps [22–24]. The majority of the studies took place in England, with the second most frequent location being in the United States.

**Table 1:** Summary of Included Studies

Author and Year	Experimental Groups	Sample Sizes and Trial Retention Rates	Lifestyle behaviors targeted	Theoretical basis of intervention	Methods of Intervention Delivery	How intervention is tailored?	Intervention Length & Contact between trial staff and participants
*Baker, 2002*	Pyscho-social intervention; Control is usual care (assessments only)	Intervention = 373 and Control = 371; 87% follow-up in intervention, 85% in control	Fruit and vegetable consumption	Stages of Change model	**Int:** Two pages of psycho-educational intervention mailed to intervention group participants; **Cntr:** Only assessments mailed to control group	Behavior change suggestions proposed in stage matched fashion that was personalized to each participant with guidance on how to overcome negative attitudes	6 weeks intervention, no contact between trial staff + participants
*Caswell, 2009*	Intervention is Bowel Health to Better Health (BHBH) group; Control is usual care (assessments only)	Intervention = 32 and Control = 30; 84% retention rate throughout intervention	Fiber intake, fruit/vegetable consumption, physical activity	Promotion of self-efficacy, personalized feedback, personalized goal setting	**Int:** One in-person meeting (2 h), and three tailored mailings for individuals in BHBH group; **Cntr:** Only completed assessments	Each mailing contained personalized goals informed by baseline data and self-efficacy for behavior change	12 week intervention, one in-person contact at Week 1
*Knudsen, 2018*	Tailored feedback (TF) group; standard leaflet (SL) group; Control group is usual care (assessments only)	Tailored feedback = 308, standard leaflet = 392, control = 354; Retention is 67.5% for TF group, 77.4% for SL group, and 76.0% for control group	Smoking, physical activity, diet, alcohol consumption,	No explicit theoretical framework applied	**Int:** Mailed leaflets that are either standard or tailored for SL and TF groups; **Cntr:** Only assessments	Data from baseline survey informed the mailed leaflet for the tailored feedback group	One year intervention, no contact between trial staff and participants outside of mailings
*Emmons, 2005*	PREVENT Intervention Group; Control is usual care (assessments only)	Intervention = 591 and Control = 656; Retention for PREVENT was 83.4% and control was 91.0%	Physical activity, fruit and vegetable consumption, multivitamin intake, red meat consumption, alcohol intake, and smoking	Social cognitive theory	**Int:** Telephone calls for coaching, mailed materials for personalized feedback and self-help information; **Cntr:** Only assessments	Counseling and mailings tailored by baseline survey data, a computer-based counseling protocol, and existing health behaviors	Three year intervention, five total contacts between health counselors and participants (baseline + 4 follow-ups)
*Mckeown-Eyssen, 1999*	Intervention is low fat high fiber (LFHF) diet; Control is normal western diet (ND)	Intervention (LFHF) = 99 and Control (ND) = 102; Retention in ND is 85.3% and in LFHF is 78.8%	Fruit/Vegetable consumption, Fiber consumption	No explicit theoretical framework applied	**Int:** Monthly in-person counseling for 12 months; **Cntr:** Only assessments	Initial food records and discussion of participants' dietary preferences over time informed counseling along with reinforcement learning strategies	12 months intervention, 12 contacts between trial administration and participants (monthly counseling)
*Robb, 2010*	Tailored feedback (TF) group; standard leaflet (SL) group; Control is usual care (assessments only)	SL = 109, TF = 103, control = 153; Retention for SL is 63.3% and 70.0% for TF and 62.7% for control	Physical activity, fruit/vegetable consumption, alcohol consumption	No explicit theoretical framework applied	**Int:** SL and TF groups received the intervention by mail approximately 9.5 weeks after screening; **Cntr:** Only assessments	Responses to baseline questionnaire informed mailed leaflet for TF group	6 months intervention, no contact between trial staff and participants outside mailings
*Wolin, 2012*	Intervention is 60 min/day high intensity walking group; Control is 30 min/day walking group;	Intervention = 8 and Control = 8; Retenion in 30-min group is 62.5% and 60-min group is 100%	Physical activity	No clear theoretical underpinning	**Int + Cntr:** Received group coaching sessions with follow-up phone calls weekly. Individuals recorded step counts via a pedometer, logged goals and step counts, and progress reports were mailed	Individual progress reports informed by participant reported step count, personalized coaching calls that contained strategy discussion and personalized goal setting	12 week intervention, 4 weekly group coaching sessions weekly and eight weekly follow-up phone calls
*Anderson, 2014*	Intervention is BeWEL coaching group; Control is usual care (assessments only)	163 to BeWEL, 166 to control; Retention is 91% BeWEL and 95% control	Fruit/Vegetable consumption, alcohol consumption, physical activity, **BMI**	No specific theory applied	**Int:** Individual counseling visits, follow-up telephone consultations using motivational interviewing techniques. Scales and pedometers given to participants to record and recall body weight and step count. **Cntr:** Only assessments	Tailored based on participants' eating habits and baseline physical activity levels/ability. Participants set their own goals and counseling focused on their experience	12 month intervention, 3 one on one visits (1 h each month), 9 telephone consultations lasting about 15 min (each month)
*Tilley, 1999*	Personalized nutrition intervention; Control is usual care (only assessments)	Intervention = 1578 and Control = 1899; 58% of participants completed assessments at all time points	Fruit and vegetable consumption, Fiber intake, Fat consumption	Social cognitive theory, social support principles, and stages of change construct	**Intr:** In-person classes, mailed self-help materials, and personalized feedback from questionnaires; **Cntr:** Only assessments	Personalized feedback from baseline questionnaires. Graphic comparison of diet to food pyramid, motivational messages during classes and feedback	24 month intervention, Five workshops in office and mailed self-help materials in first 12 months, and worksite posters and personalized feedback in second 12 months
*Lewis, 2020*	Physical activity (PA) intervention; Control is usual care (assessments only)	Intervention = 17 and Control = 14; 6 month retention = 70.6% for PA intervention and 58.8% for control; 12 month retention = 47.1% for PA intervention and 50% for control	Physical Activity, **BMI**	Self-determination theory	**Intr:** Supervised group exercise sessions delivered along with bi-weekly behavior change workshops to aid the uptake and maintenance of physical activity; **Cntr:** Only assessments	Exercise program tailored to individual capabilities to maintain an adequate stimulus for adaptation and autonomy. Behavior workshops utilized motivational interviewing	48 total interactions across 6 months. 36 supervised exercise sessions (30 min aerobic exercise @ 65–85% MHR plus 10–15 min RT, 1–2 × /week) plus 12 weekly behavior change workshops

Seven of the studies addressed more than one specific lifestyle behavior, while three focused on only either fruit/vegetable consumption or physical activity. The median length of interventions was nine months with a range of 1.5 to 36 months. The median intervention sample size was 347 with the range being 16 to 3477.

“Int” refers to the intervention group and “Cntr” refers to the control group within a randomized controlled trial.

### Risk of bias

**Fig. 3:**
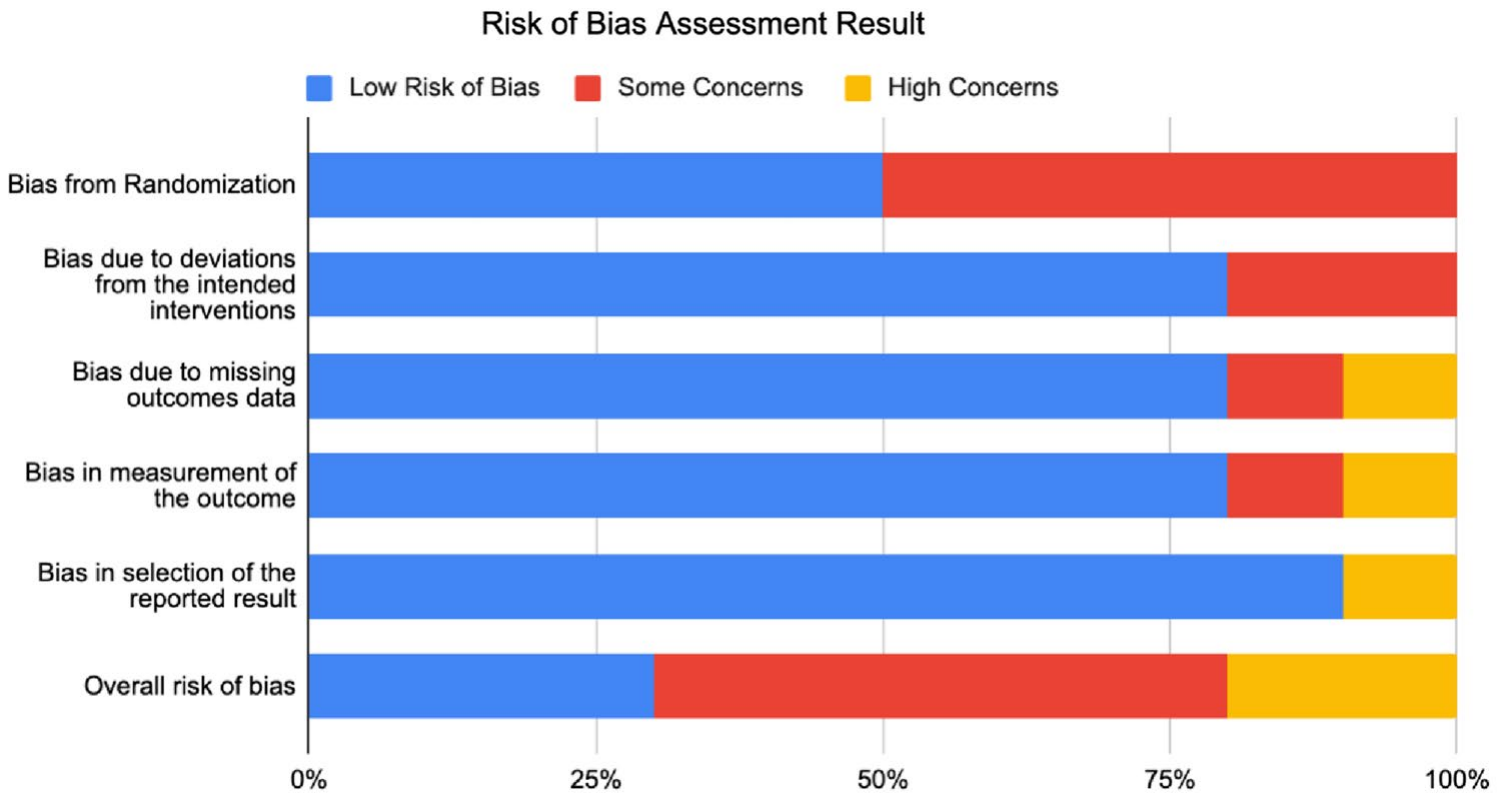
Distribution of bias across the five ROB2 sub-domains

Of the ten studies included in this review, three were determined to have low risk of bias, five were determined to have some concerns, and two were determined to have high risk of bias. The greatest source of bias in the included studies was bias from randomization, as nearly half of the included studies did not apply specific allocation sequences or computer-generated programs for randomizing participants. Judgements on each of the five domains of potential bias as per the ROB2 tool are shown below in Fig. 3.

### Outcomes

**Fig. 4:**
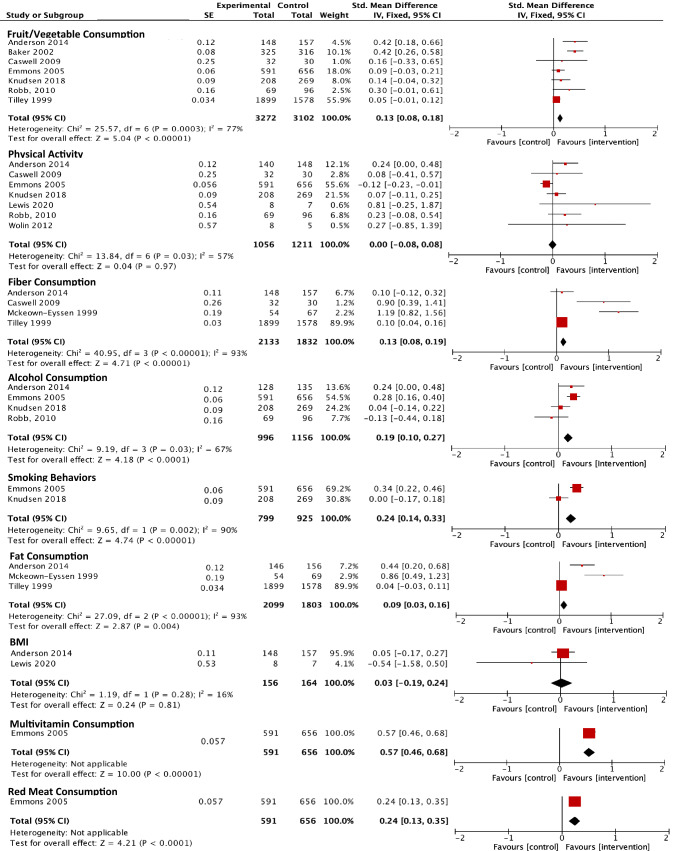
Standardized mean differences and pooled effect sizes across outcomes of interest

The results of the meta-analysis for each outcome are shown in Fig. 4.

#### Fruit/Vegetable consumption

Fruit/vegetable consumption was measured in seven of the ten included studies. To measure fruit/vegetable consumption, studies used the Dietary Instrument for Nutrition Education (DINE) [9, 20], the National Cancer Institute Food Frequency Questionnaire (NCI FFQ) [26], the Norwegian LSQ instrument [8], and a two-item questionnaire by *Carpuccio *et al*.* [21]. Pooled data from the seven included studies showed a greater increase in fruit/vegetable consumption in the intervention group with respect to the control (SMD 0.13, 95% CI 0.08 to 0.18; *p* < 0.001).

#### Physical activity

Similar to fruit/vegetable consumption, seven of the ten included studies measured physical activity. To measure physical activity, studies used accelerometry and pedometer data [21, 22, 24], and self-report measures including the Scottish Physical Activity Questionnaire [20], the Norwegian LSQ [8], the CHAMPS questionnaire [25], and the International Physical Activity Questionnaire (IPAQ) [22]. Our meta-analysis showed no difference in physical activity between the intervention groups compared with controls.

#### Fiber consumption

Four studies reported outcomes on fiber consumption. To measure fiber consumption, studies used biochemical testing of stool samples [23] and both the DINE [20, 21] and NCI FFQ instruments [26]. The DINE fiber scores differ from the DINE score for fruit/vegetable consumption and ranges from 3 to 88 (arbitrary units) with a score of less than 30 (low) corresponding to a fiber intake of ≤ 20 g/day, and a score of more than 40 (high) corresponding to ≥ 30 g/day. Pooled data from the four included studies showed a greater increase in fiber consumption in the intervention group with respect to the control (SMD 0.14, 95% CI 0.08 to 0.20; *p* < 0.001).

#### Alcohol consumption

Four studies reported outcomes on alcohol consumption. Included studies measured alcohol consumption via self-report measures such as the NCI FFQ [25], Norwegian LSQ [8], and the Alcohol Use Disorders Inventory Test [21]. Pooled data from the four included studies showed a greater decrease in alcohol consumption in the intervention group with respect to the control group (SMD 0.19, 95% CI 0.11 to 0.27; *p* < 0.001).

#### Smoking behaviors

Two studies reported outcomes on smoking behaviors. Similar to measurements on alcohol consumption behaviors, smoking behaviors were measured via self-report using the NCI FFQ [25] and Norwegian LSQ [8]. Data from the two studies showed a showed a greater proportion of individuals not smoking in the intervention group with respect to the control group (SMD 0.24, 95% CI 0.15 to 0.34; *p* < 0.001).

#### Fat consumption

Three studies reported outcomes on fat consumption. Fat consumption was measured through self-report via the NCI FFQ and DINE instruments [21, 26], or via nutrient analysis of stool samples [23]. Pooled data from the three included studies showed a greater decrease in fat consumption in the intervention group with respect to the control, however, the strength of the effect size is smaller than that of other outcomes (SMD 0.09, 95% CI 0.03 to 0.15; *p* = 0.004).

#### Body mass index

Two studies reported outcomes body weight. Body mass index was measured through either self-reported or research team measured weight in kilograms and height in centimeters. Pooled data from the two included studies showed no difference in body between the intervention groups compared with controls (SMD 0.03, 95% CI -0.19 to 0.24; *p* = 0.81).

#### Multivitamin consumption + red meat consumption

Multivitamin and red meat consumption were each measured by one study. Multivitamin consumption was measured via a single-item self-reported response modified from the Nurse’s Health Study [25], and the SMD of 0.53 indicates a moderate effect size given *Cohen’s* criteria. Red meat consumption was measured by the semi-quantitative FFQ [25] and had a low effect size with an SMD of 0.24 between those in the intervention group and those in the control group.

#### Heterogeneity across outcomes

Across all outcomes of interest, we saw moderate to high between-study heterogeneity, evaluated through the I^2^ value. Between-study heterogeneity varied from 46% among studies measuring alcohol consumption to those measuring fiber consumption and fat consumption, both of which were 93%. Specific values for each outcome are shown below in Fig. 4.

## Application of behavior change techniques

**Fig. 5:**
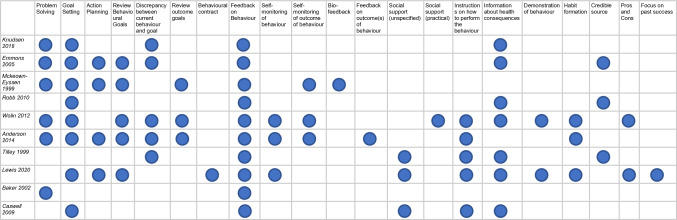
Frequency of specific BCT’s applied across the included studies

Each included study leveraged behavior change techniques from the *Michie *et al*.* taxonomy [15]. As shown in Fig. 5, the median number of BCTs applied by the included studies was 7.5, with a range of two to 13. The most common BCTs applied across the interventions were “feedback on behaviour,” “goal setting,” “information about health consequences,” and “review behavioural goals.”

The application of behavior change techniques was far more common among interventions with in-person coaching or demonstration components. For example, each intervention with more than 10 BCTs applied had one-on-one or group coaching services offered during the intervention. Additionally, interventions that reinforced mechanisms of habit tracking, such as self-weighing and pedometer use [21, 22, 24], applied more BCTs than interventions that solely focused on didactic learning. Interventions which used leaflets/mailings as the primary form of delivery applied the fewest BCTs.

While a meta-regression was not completed in this study, we observed no consistent pattern between the number of behavior change techniques applied and the effect sizes of the studies across our outcomes of interest.

## Discussion

Screening programs have been shown to be an effective means of reducing CRC disease burden, and a growing body of literature has described colorectal cancer screening as a “teachable moment” for behavior change [7, 27, 28]. Our findings suggest that interventions seeking to change behaviors related to diet, physical activity, alcohol consumption, and smoking achieved positive, albeit modest, improvements in these behaviors compared with usual care. We additionally no found evidence that a greater number of BCTs was associated with greater behavior change.

While improvements in lifestyle behavior uptake at the individual level were modest, they represent potentially important differences at a population level. For example, the pooled effect size of 0.13 for fruit/vegetable consumption corresponds to an additional 0.3 – 0.6 servings of fruits/vegetables per day [20, 21]. At a population level, if sustained, this increase in fruit/vegetable consumption is estimated to be associated with between a 3 and 6% reduction in CRC risk [29]. Similarly, the pooled effect size for fiber consumption of 0.13 corresponds to a 1.6 g increase in daily fiber intake. Given that each 10 g of fiber consumed daily results in a 7% reduction in CRC risk, this change in behavior, if sustained, corresponds to a 1.12% decrease in population-wide CRC risk [30]. We found similar results for consumption of alcohol and fat. An effect size of 0.09 for fat consumption corresponds to a reduction in daily fat intake of approximately 2.1 g [21, 23]. Given the instruments used, it was not possible to compare pooled effect sizes for alcohol consumption.

Effect sizes for smoking**,** multivitamin consumption, and red meat consumption similarly all favored the intervention groups. However, the number of studies measuring each outcome was fewer than that for other pooled outcomes. Nevertheless, these results are also potentially promising at the population level. Most notably, previous literature from the World Health Organization suggests that eating 50 g of processed meat daily increases the risk of colorectal cancer by 18% [31], and *Botteri et. al* suggests that regular smoking increases CRC risk by 15–20% [32].

Although some interventions demonstrated moderate increases in physical activity compared with the respective controls, particularly the three studies including self-monitoring [21, 22, 24], overall the pooled effect sizes show no evidence of change in physical activity. One potential reason for this is the lack of self-monitoring processes included within the other interventions, which were shown to be critical to physical activity behavior change across other interventions. The lack of self-monitoring across interventions is demonstrated in Fig. 4 where it is applied in only three studies [33]. Similar results were concluded for body mass index, which showed no evidence of change even though it has been identified as a risk factor for CRC. Future interventions should further consider how to address body mass index and body weight given its association with CRC risk, and additionally measure body mass index as an outcome especially among studies addressing diet and physical activity.

Previous literature has linked the application of behavior change techniques to positive lifestyle behavior change among individuals eligible for CRC screening [33–37]. Within this study, a meta-regression was not possible due to the limited number of studies, so we were unable to directly quantify the association between applying BCTs and the effectiveness of interventions. However, we found that interventions with a greater number of in-person contacts and tailored information provision applied a greater number of BCTs. The BCTs most frequently utilized among included studies, such as feedback on behavior, goal setting, problem solving, and information on health consequences, have been commonly applied in lifestyle behavior change interventions outside of CRC screening [33, 38]. Future interventions exploring behavior change within this context should explore applying these specific BCTs. However, as we identified no direct relationship between the application of BCTs and the observed effect sizes, additional work is needed to explore the value of BCTs specifically within the context of CRC screening [33–38].

Given the BCTs identified within this review, one avenue to further promote lifestyle behavior change within the context of CRC screening is to increase both the level of participant engagement and the effectiveness of existing engagement within interventions [39, 40]. One avenue may be the application of digital components or tools, especially given the existing literature supporting the effectiveness of such interventions [41, 42]. For example, a number of publications have cited the benefit of applying digital tools to improve the frequency, duration, and tailoring of engagement along with the depth of engagement within behavior change interventions [43–45]. However, within the context of CRC screening, additional considerations must be made to the effect of participant age within digital interventions. While digital interventions applying BCT’s focused on older populations have shown initial success, further evidence is needed to draw conclusions surrounding efficacy [47–49]. Specifically, opportunities to engage patients over longer periods of time as means to promote sustained behavior change are needed, especially those that can achieve outcomes in a cost-effective manner.

The findings of this systematic review have significant relevance among the broader body of literature promoting lifestyle behavior change as a means of reducing cancer risk. Across several lifestyle behaviors, this review quantified the impact of specific interventions on CRC risk, which while small in magnitude, are significant on a population level. *Orange *et al. highlighted similar conclusions across both colorectal and breast cancer, and numerous other studies have shown the association between lifestyle behaviors such as diet, physical activity, smoking, and red meat consumption and cancer risk [50–55]. However, additional work is needed applying similar interventions to other cancers, and secondarily contextualizing the value of screening as an opportunity for health promotion.

This review has several strengths that build on limitations of previous reviews investigating the effectiveness of behavior change interventions implemented during CRC screening. First, all articles meeting initial database search criteria were double screened. Second, this review includes interventions whose participants have undergone previous investigations for CRC, allowing us to evaluate interventions that may be applicable to the context of screening itself. This is especially relevant within the context of national CRC screening programs, which have multiple stages and thus require longer-term engagement across heterogeneous patient population. Furthermore, characterizing the BCTs applied across interventions provides valuable insight into how future interventions could better integrate behavioral science principles to target specific behavior change outcomes. Additionally, the majority of interventions within this review (7/10) had clinical professionals involved with intervention delivery, indicating a focus on implementation within clinical practice.

Additionally, this review has several limitations. Within each outcome in the meta-analysis, we observed moderate to high levels of between-study heterogeneity, and 70% of the included studies had “some concerns” or “high risk” of bias as per the Cochrane ROB2 tool primarily due to the lack of robust randomization methods applied. The between-study heterogeneity may be due to the fact that many of the primary measurement strategies across these outcomes were self-report and recall measures of limited rigor with different lengths of follow-up measurements. For example, to measure physical activity, several studies used less rigorous self-report measures as compared to more objective measures such as pedometer data [21, 22, 24]. To reduce bias and error, future interventions measuring change in lifestyle behaviors should seek to implement more objective measurement strategies. Many of the studies included in this review were conducted either in the United Kingdom or United States, and as such, the results may also not be directly applicable to the contexts of other healthcare systems around the world. Three studies measured outcomes for less than 6 months, limiting our understanding of the long-term effectiveness of such interventions. Lastly, we only included studies in English in this review, which may limit the identification of several studies in gray literature and additional peer-reviewed literature in other languages.

## Conclusion

In this systematic review we have identified positive improvements in fruit/vegetable consumption, fiber consumption, fat consumption, red meat and multivitamin consumption, alcohol consumption, and smoking behaviors following interventions either delivered within CRC screening program or targeted specifically at reducing CRC risk among those eligible for CRC screening. While the magnitude of the pooled effect sizes varied, they correspond to potentially important changes in lifestyle behaviors when considered on a population scale. Interventions with more in-person engagement between participants and trial staff applied a greater number of BCTs, however we saw no pattern between the number of BCTs applied and the observed effect size. Together with evidence from previous work, our findings indicate that interventions delivered at the time of screening or shortly afterward represents a promising opportunity to reduce CRC risk through behavior change. Future interventions should identify avenues to maximize long-term engagement to promote sustained lifestyle behavior change.
